# Redetermination of cytosinium hydrogen maleate–cytosine (1/1) from the original data

**DOI:** 10.1107/S2056989016003923

**Published:** 2016-03-15

**Authors:** Jan Fábry

**Affiliations:** a Inst. of Physics of the Czech Academy of Sciences, Na Slovance 2, 182 21 Praha 8, Czech Republic

**Keywords:** crystal structure, redetermination, hydrogen bonding, refinement constraints, refinement restraints, Cambridge Structural Database

## Abstract

The title salt, C_4_H_6_N_3_O^+^·C_4_H_3_O_4_^−^·C_4_H_5_N_3_O, has been redetermined from the data published by Benali-Cherif, Falek & Direm [*Acta Cryst.* (2009), E**65**, o3058–o3059]. The improvement of the present redetermination consists in the discovery of the splitting of one of the H atoms into two disordered positions, the occupancies of which are equal to 0.55 (2) and 0.45 (2). These H atoms are involved in an N⋯N hydrogen bond and are shifted towards its centre. The disorder of these H atoms is in agreement with a similar environment of the two independent, but chemically equivalent, cytosinium/cytosine mol­ecules.

## Abstract 2

The title structure has been redetermined from the data published by Benali-Cherif, Falek & Direm [*Acta Cryst.* (2009), E**65**, o3058–o3059]. The improvement of the present redetermination consists in the discovery of the disorder of one of the H atoms with occupancies equal to 0.55 (2) and 0.45 (2), respectively. These H atoms are involved in an N⋯N hydrogen bond and are shifted towards its centre.

## Chemical context   

**Figure 1:**
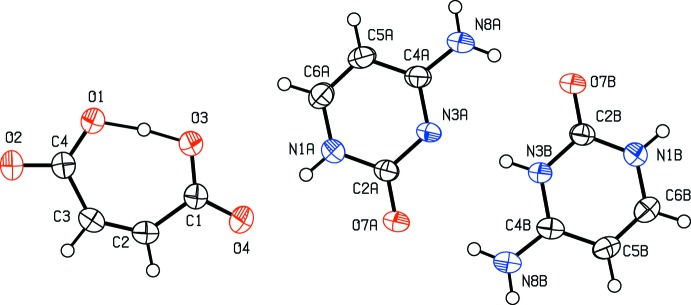
View of the constituent mol­ecules and atoms of the title structure in the original article [Benali-Cherif, Falek & Direm (2009 ▸). *Acta Cryst.* E**65**, o3058–o3059]. The displacement ellipsoids are drawn at the 50% probability level.

Structures which contain hydroxyl, secondary and primary amine groups are often determined incorrectly because of an assumed geometry of these groups and the subsequent applied constraints or restraints. In such cases, the correct geometry is missed as it is not verified by inspection of the difference electron-density maps. Thus a considerable number of structures could have been determined more correctly – *cf.* Figs. 1 ▸ and 2 ▸ in Fábry *et al.* (2014 ▸). The inclusion of such structures causes bias in the crystallographic databases.
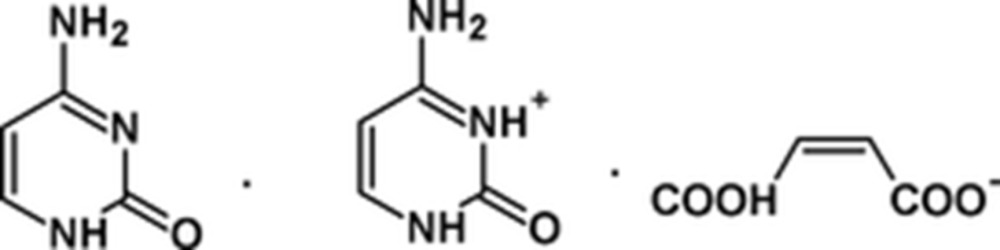


In the course of recalculation of suspect structures which were retrieved from the Cambridge Crystallographic Database (Groom & Allen, 2014 ▸), a defect in the structure determination of 2-amino-4,6-di­meth­oxy­pyrimidine–4-amino­benzoic acid (1/1) by Benali-Cherif *et al.* (2009 ▸) has been found; the CSD refcode is *DUJCAN*. The aim of the present article is to demonstrate how the original structure determination can be improved.

## Structural commentary   

The structure of the title compound has been described by Benali-Cherif *et al.* (2009 ▸). In that article, the hydrogen atom H3*b* was attached to atom N3*b* and refined with a distance constraint of N3*b*—H3*b* = 0.86 Å with *U*_iso_(H3*b*) = 1.2*U*_eq_(N3*b*). This hydrogen is involved in the hydrogen bond N3*b*—H3b⋯N3*a* (Fig. 1 ▸).

**Figure 2:**
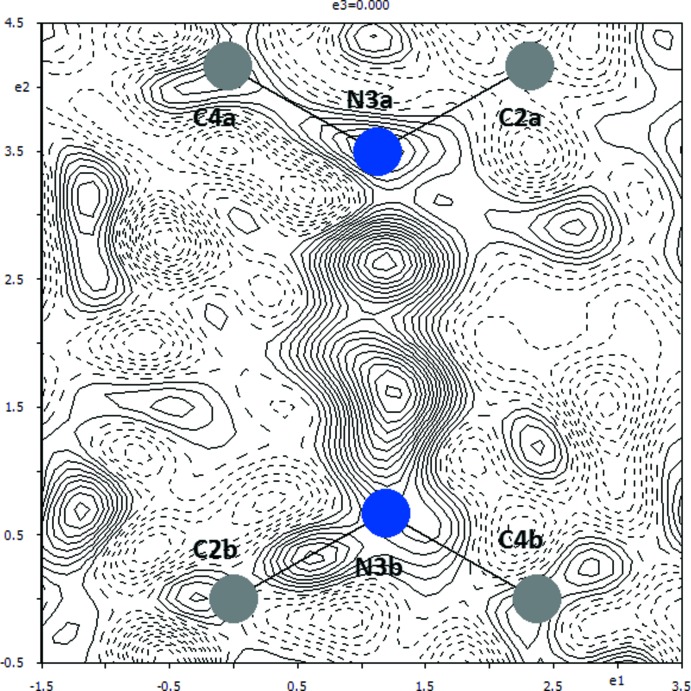
A section of the difference electron-density map for the present redetermined title structure, which shows the build up of the electron density between atoms N1 and N3. Positive and negative electron densities are indicated by continuous and dashed lines, respectively. The increment between the contours is 0.05 e Å^−3^ (*JANA*2006; Petříček *et al.*, 2014 ▸).

**Figure 3:**
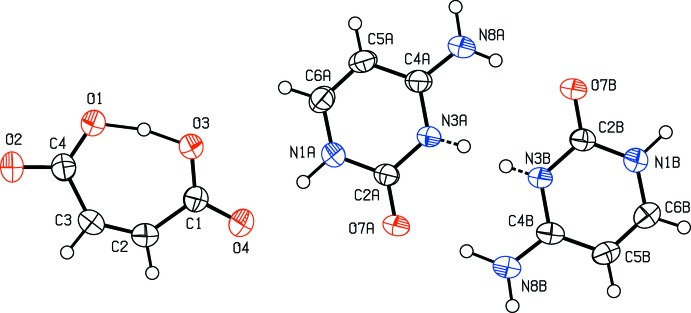
View of the constituent mol­ecules and atoms of the present redetermined title structure. The displacement ellipsoids are drawn at the 50% probability level.

However, inspection of the difference electron density map of the recalculated structure has shown that hydrogen atom H3*b* is disordered over two positions (Fig. 2 ▸), between atoms N3*a* and N3*b*. Thus, atom H3*b* was split into two atoms, labelled as H1*n*3*b* and H1*n*3*a*, with respective occupancies 0.52 (2) and 0.48 (2). These hydrogen atoms remain involved in the N3*a*⋯N3*b* hydrogen bond (Table 1 ▸), as shown in Fig. 3 ▸.

The observed disorder of the secondary amine hydrogen atoms is probably due to the chemical equality of two symmetry-independent cytosinium/cytosine mol­ecules and their quite similar environments. Otherwise, the description of the hydrogen-bond pattern by Benali-Cherif *et al.* (2009 ▸) remains intact because locally one of the nitro­gen atoms, N3*a* or N3*b*, acts as a donor while the other acts as an acceptor of the hydrogen bond.

**Figure 4:**
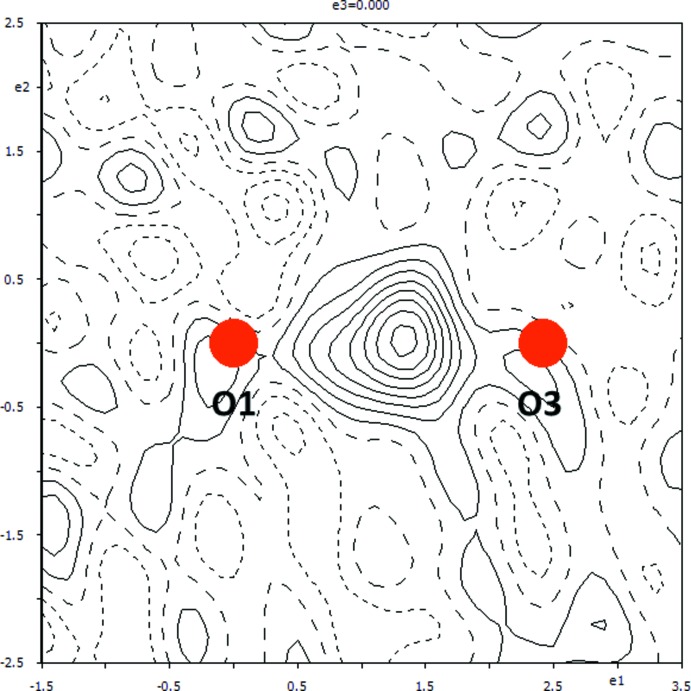
A section of the difference electron-density map for the present redetermined title structure, which shows the build up of the electron density between atoms O1 and O3. Positive and negative electron densities are indicated by continuous and dashed lines, respectively. The increment between the contours is 0.05 e Å^−3^ (*JANA*2006; Petříček *et al.*, 2014 ▸).

The hydrogen atom H3, which was situated about the centre of the hydrogen bond O3—H3⋯O1 has also been checked (Fig. 4 ▸). It turns out that the build-up of the electron density is not split into two positions and the original position determined by Benali-Cherif *et al.* (2009 ▸) is correct.

In a broader sense, the present redetermination emphasizes how important it is to carefully examine the difference electron-density maps during structure determinations.

## Supra­molecular features   

The graph set analysis (Etter *et al.*, 1990 ▸) of the title compound has been described by Benali-Cherif *et al.* (2009 ▸).

## Database survey   

The CIF file of the article by Benali-Cherif *et al.* (2009 ▸) has been included in the Cambridge Crystallographic Database (Groom & Allen, 2014 ▸) under the refcode *DUJCAN*.

## Synthesis and crystallization   

The preparation of the title compound has been described by Benali-Cherif *et al.* (2009 ▸).

## Refinement   

Crystal data, data collection and structure refinement details are summarized in Table 2 ▸. All the hydrogen atoms were discernible in the difference electron density maps. The aryl hydrogen atoms were refined as constrained with C_ar­yl_—H_ar­yl_ = 0.93 Å and *U*_iso_(H_ar­yl_) = 1.2*U*_eq_(C_ar­yl_). The displacement parameter of the hydroxyl hydrogen atom H3 was constrained by *U*_iso_(H3) = 1.5*U*_eq_(O3). The hydrogen atoms of the primary and secondary amine groups were constrained by *U*_iso_(H_amine_) = 1.2*U*_eq_(N_amine_). In addition, the distances of the disordered amine hydrogen atoms, H1*n*36 and H1*n*3*b*, were refined with the distance restraint N—H = 0.87 (1) Å, and their occupational parameters constrained to fulfill the condition that their sum = 1 [*viz*. 0.55 (2) (H1*n*3*b*) and 0.45 (2) (H1*n*3a)].

Nine reflections [5 1 0; −9 1 1;-1 1 1; −8 2 1; 4 2 1; −2 0 2; 0 0 2;-3 1 2; −20 0 8; 22 2 8] for which ||*F*_o_| − |*F*_c_|| >10σ(*F*) were omitted from the final cycles of refinement.

## Supplementary Material

Crystal structure: contains datablock(s) global, I. DOI: 10.1107/S2056989016003923/su5280sup1.cif

Structure factors: contains datablock(s) I. DOI: 10.1107/S2056989016003923/su5280Isup2.hkl

Supporting information file. DOI: 10.1107/S2056989016003923/su5280Isup3.smi

CCDC reference: 1459296

Additional supporting information:  crystallographic information; 3D view; checkCIF report

## Figures and Tables

**Table 1:** Hydrogen-bond geometry (Å, °)

*D*—H⋯*A*	*D*—H	H⋯*A*	*D*⋯*A*	*D*—H⋯*A*
N1*b*—H1*b*⋯O2^i^	0.956 (15)	1.824 (15)	2.7718 (14)	170.8 (13)
N8*b*—H8*b*1⋯O7*b* ^ii^	0.900 (17)	2.030 (18)	2.8517 (14)	151.2 (13)
N8*b*—H8*b*2⋯O7*a*	0.992 (15)	1.850 (15)	2.8411 (15)	177.4 (12)
C5*b*—H5*b*⋯O2^iii^	0.93	2.43	3.3347 (16)	164.60
N1*a*—H1*a*⋯O4	0.952 (14)	1.793 (14)	2.7411 (14)	173.2 (12)
N8*a*—H8*a*1⋯O7*b*	0.897 (16)	1.959 (16)	2.8555 (15)	179.0 (13)
N8*a*—H8*a*2⋯O7*a* ^iv^	0.885 (17)	2.028 (18)	2.8368 (15)	151.5 (14)
C5*a*—H5*a*⋯O4^iv^	0.93	2.37	3.2970 (16)	175.15
O1—H3⋯O3	1.223 (14)	1.201 (14)	2.4155 (12)	170.6 (15)
O1—H3⋯C1	1.223 (14)	2.071 (15)	3.0775 (15)	136.7 (11)
O3—H3⋯O1	1.201 (14)	1.223 (14)	2.4155 (12)	170.6 (15)
O3—H3⋯C4	1.201 (14)	2.100 (15)	3.0927 (15)	137.4 (12)
N3*b*—H1*n*3*b*⋯N3*a*	0.861 (16)	1.979 (16)	2.8398 (14)	178 (2)
N3*a*—H1*n*3*a*⋯N3*b*	0.873 (18)	1.970 (18)	2.8398 (14)	174 (3)

Symmetry codes: (i) 
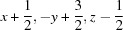
; (ii) 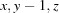; (iii) 
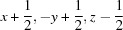
; (iv) 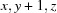.

**Table 2:** Experimental details

Crystal data	
Chemical formula	C_4_H_6_N_3_O^+^·C_4_H_3_O_4_^−^·C_4_H_5_N_3_O
*M*_r_	338.29
Crystal system, space group	Monoclinic, *C*2/*c*
Temperature (K)	298
*a*, *b*, *c* (Å)	27.3226 (5), 7.3618 (2), 14.6742 (4)
β (°)	93.905 (1)
*V* (Å^3^)	2944.77 (13)
*Z*	8
Radiation type	Mo *K*α
μ (mm^−1^)	0.13
Crystal size (mm)	0.3 × 0.15 × 0.1

Data collection	
Diffractometer	Nonius KappaCCD
No. of measured, independent and observed [*I* > 3σ(*I*)] reflections	3490, 3474, 2367
*R*_int_	0.043
(sin θ/λ)_max_ (Å^−1^)	0.661

Refinement	
*R*[*F* ^2^ > 3σ(*F* ^2^)], *wR*(*F* ^2^), *S*	0.038, 0.093, 1.85
No. of reflections	3474
No. of parameters	246
No. of restraints	2
H-atom treatment	H atoms treated by a mixture of restrained and constrained refinement
Δρ_max_, Δρ_min_ (e Å^−3^)	0.20, −0.20

Computer programs: *KappaCCD Server Software* (Nonius, 1998 ▸), *DENZO* and *SCALEPACK* (Otwinowski & Minor, 1997 ▸), *SIR2004* (Burla *et al.*, 2005 ▸), *PLATON* (Spek, 2009 ▸) and *JANA*2006 (Petříček *et al.*, 2014 ▸). Extinction correction: Becker & Coppens (1974 ▸).

## supplementary crystallographic information

### Crystal data

**Table:**

C_4_H_6_N_3_O^+^·C_4_H_3_O_4_^−^·C_4_H_5_N_3_O	*F*(000) = 1408
*M**_r_* = 338.29	*D*_x_ = 1.526 Mg m^−^^3^
Monoclinic, *C*2/*c*	Mo *K*α radiation, λ = 0.71073 Å
Hall symbol: -C 2yc	Cell parameters from 3490 reflections
*a* = 27.3226 (5) Å	θ = 2.8–28.0°
*b* = 7.3618 (2) Å	µ = 0.13 mm^−^^1^
*c* = 14.6742 (4) Å	*T* = 298 K
β = 93.905 (1)°	Prism, colourless
*V* = 2944.77 (13) Å^3^	0.3 × 0.15 × 0.1 mm
*Z* = 8	

### Data collection

**Table:**

Nonius KappaCCD diffractometer	2367 reflections with *I* > 3σ(*I*)
Radiation source: fine-focus sealed tube	*R*_int_ = 0.043
Graphite monochromator	θ_max_ = 28.0°, θ_min_ = 2.8°
ω–θ scans	*h* = 0→35
3490 measured reflections	*k* = 0→9
3474 independent reflections	*l* = −19→19

### Refinement

**Table:**

Refinement on *F*^2^	H atoms treated by a mixture of independent and constrained refinement
*R*[*F*^2^ > 2σ(*F*^2^)] = 0.038	Weighting scheme based on measured s.u.'s *w* = 1/(σ^2^(*I*) + 0.0004*I*^2^)
*wR*(*F*^2^) = 0.093	(Δ/σ)_max_ = 0.022
*S* = 1.85	Δρ_max_ = 0.20 e Å^−^^3^
3474 reflections	Δρ_min_ = −0.20 e Å^−^^3^
246 parameters	Extinction correction: B–C type 1 Lorentzian isotropic (Becker & Coppens, 1974)
2 restraints	Extinction coefficient: 21000 (5000)
33 constraints	

### Special details

**Table:**

Refinement. This part differs from the original article by Benali-Cherif *et al.* (2009). In the refinement, *F*^2^ > 3σ(*F*^2^) has been used as a criterion for observed diffractions.The diffractions for which ||*F_o_*|-|Fc||>10σ(*F*) were discarded from the refinement. This refers to the diffractions 5 1 0; −9 1 1; −1 1 1; −8 2 1; 4 2 1; −2 0 2; 0 0 2; −3 1 2; −20 0 8; 22 2 8.

### Fractional atomic coordinates and isotropic or equivalent isotropic displacement parameters (Å^2^)

**Table:**

	*x*	*y*	*z*	*U*_iso_*/*U*_eq_	Occ. (<1)
O7b	0.33926 (3)	1.06385 (11)	0.29287 (6)	0.0445 (3)	
N1b	0.40065 (3)	0.90018 (14)	0.23744 (7)	0.0400 (3)	
H1b	0.4157 (5)	1.011 (2)	0.2197 (9)	0.048*	
N3b	0.33591 (3)	0.75686 (12)	0.30318 (7)	0.0368 (3)	
N8b	0.33297 (5)	0.44786 (15)	0.31476 (9)	0.0506 (4)	
H8b1	0.3454 (5)	0.338 (2)	0.3026 (9)	0.0607*	
H8b2	0.3002 (6)	0.458 (2)	0.3394 (9)	0.0607*	
C2b	0.35770 (4)	0.91429 (16)	0.27860 (8)	0.0352 (4)	
C4b	0.35664 (4)	0.59317 (16)	0.28928 (8)	0.0386 (4)	
C5b	0.40187 (4)	0.58331 (18)	0.24835 (9)	0.0443 (4)	
H5b	0.416945	0.472174	0.23913	0.0531*	
C6b	0.42233 (5)	0.73892 (18)	0.22326 (9)	0.0443 (4)	
H6b	0.451959	0.735942	0.195619	0.0531*	
O7a	0.23772 (3)	0.47438 (12)	0.38018 (7)	0.0481 (3)	
N1a	0.17576 (3)	0.63613 (15)	0.43512 (7)	0.0413 (3)	
H1a	0.1595 (4)	0.528 (2)	0.4518 (9)	0.0496*	
N3a	0.24303 (3)	0.78185 (13)	0.37815 (7)	0.0375 (3)	
N8a	0.24808 (5)	1.09110 (16)	0.37611 (9)	0.0522 (4)	
H8a1	0.2767 (6)	1.0812 (19)	0.3501 (10)	0.0627*	
H8a2	0.2363 (5)	1.200 (2)	0.3886 (10)	0.0627*	
C2a	0.21958 (4)	0.62341 (16)	0.39695 (8)	0.0364 (4)	
C4a	0.22340 (4)	0.94481 (16)	0.39665 (8)	0.0388 (4)	
C5a	0.17788 (4)	0.95478 (18)	0.43679 (9)	0.0439 (4)	
H5a	0.164021	1.066081	0.450387	0.0527*	
C6a	0.15539 (5)	0.79794 (18)	0.45457 (9)	0.0447 (4)	
H6a	0.125322	0.800462	0.480683	0.0536*	
O1	0.00024 (3)	0.51326 (12)	0.62974 (6)	0.0463 (3)	
O2	−0.05067 (3)	0.30108 (13)	0.67262 (7)	0.0560 (3)	
O3	0.07413 (3)	0.53082 (11)	0.54869 (6)	0.0433 (3)	
H3	0.0368 (5)	0.535 (2)	0.5874 (9)	0.0649*	
O4	0.12217 (3)	0.33900 (14)	0.48088 (7)	0.0555 (3)	
C1	0.08603 (4)	0.37076 (18)	0.52453 (8)	0.0394 (4)	
C2	0.05595 (5)	0.21144 (19)	0.54864 (10)	0.0512 (5)	
H1	0.067397	0.100138	0.528962	0.0614*	
C3	0.01546 (5)	0.20204 (19)	0.59362 (10)	0.0523 (5)	
H2	0.003281	0.085236	0.600501	0.0628*	
C4	−0.01355 (4)	0.34770 (18)	0.63476 (8)	0.0415 (4)	
H1n3b	0.3079 (5)	0.763 (3)	0.3267 (14)	0.0442*	0.554 (16)
H1n3a	0.2718 (6)	0.783 (4)	0.3556 (18)	0.045*	0.446 (16)

### Atomic displacement parameters (Å^2^)

**Table:**

	*U*^11^	*U*^22^	*U*^33^	*U*^12^	*U*^13^	*U*^23^
O7b	0.0462 (5)	0.0256 (5)	0.0628 (6)	0.0015 (3)	0.0110 (4)	−0.0015 (4)
N1b	0.0413 (6)	0.0342 (6)	0.0454 (6)	−0.0014 (4)	0.0100 (5)	−0.0016 (5)
N3b	0.0375 (5)	0.0249 (6)	0.0486 (6)	0.0009 (4)	0.0073 (4)	−0.0018 (4)
N8b	0.0551 (7)	0.0278 (6)	0.0705 (8)	0.0025 (5)	0.0171 (6)	0.0000 (5)
C2b	0.0374 (6)	0.0291 (7)	0.0388 (7)	0.0009 (5)	0.0007 (5)	−0.0022 (5)
C4b	0.0450 (6)	0.0285 (7)	0.0420 (7)	0.0019 (5)	0.0002 (5)	−0.0021 (5)
C5b	0.0452 (7)	0.0368 (8)	0.0516 (8)	0.0105 (5)	0.0093 (6)	−0.0029 (6)
C6b	0.0425 (6)	0.0435 (8)	0.0476 (8)	0.0062 (5)	0.0093 (6)	−0.0040 (6)
O7a	0.0468 (5)	0.0246 (5)	0.0740 (6)	0.0004 (4)	0.0134 (4)	−0.0008 (4)
N1a	0.0382 (5)	0.0335 (6)	0.0532 (6)	−0.0044 (4)	0.0100 (5)	−0.0018 (5)
N3a	0.0354 (5)	0.0246 (5)	0.0531 (6)	0.0008 (4)	0.0079 (4)	−0.0001 (4)
N8a	0.0515 (6)	0.0263 (6)	0.0806 (9)	0.0013 (5)	0.0171 (6)	0.0004 (6)
C2a	0.0374 (6)	0.0269 (7)	0.0448 (7)	0.0008 (5)	0.0024 (5)	0.0000 (5)
C4a	0.0400 (6)	0.0296 (7)	0.0467 (7)	0.0024 (5)	0.0009 (5)	−0.0017 (5)
C5a	0.0421 (7)	0.0341 (7)	0.0560 (8)	0.0085 (5)	0.0059 (6)	−0.0059 (6)
C6a	0.0375 (6)	0.0457 (8)	0.0514 (8)	0.0032 (5)	0.0073 (6)	−0.0065 (6)
O1	0.0411 (5)	0.0411 (6)	0.0582 (6)	−0.0016 (4)	0.0155 (4)	−0.0041 (4)
O2	0.0471 (5)	0.0532 (6)	0.0703 (7)	−0.0059 (4)	0.0234 (5)	0.0033 (5)
O3	0.0426 (5)	0.0365 (5)	0.0521 (5)	−0.0035 (4)	0.0137 (4)	−0.0025 (4)
O4	0.0547 (5)	0.0476 (6)	0.0677 (6)	−0.0001 (4)	0.0288 (5)	−0.0052 (5)
C1	0.0402 (6)	0.0388 (7)	0.0398 (7)	0.0003 (5)	0.0070 (5)	−0.0002 (6)
C2	0.0545 (8)	0.0338 (8)	0.0675 (9)	0.0020 (6)	0.0202 (7)	−0.0028 (6)
C3	0.0544 (8)	0.0332 (8)	0.0714 (10)	−0.0045 (6)	0.0185 (7)	0.0033 (7)
C4	0.0383 (6)	0.0417 (8)	0.0449 (7)	−0.0028 (5)	0.0059 (5)	0.0025 (6)

### Geometric parameters (Å, º)

**Table:**

O7b—C2b	1.2345 (14)	N3a—H1n3a	0.873 (18)
N1b—H1b	0.956 (15)	N8a—H8a1	0.897 (16)
N1b—C2b	1.3600 (15)	N8a—H8a2	0.885 (17)
N1b—C6b	1.3492 (17)	N8a—C4a	1.3165 (17)
N3b—C2b	1.3627 (15)	H8a1—H8a2	1.54 (2)
N3b—C4b	1.3528 (15)	C4a—C5a	1.4137 (17)
N3b—H1n3b	0.861 (16)	C5a—H5a	0.93
N8b—H8b1	0.900 (17)	C5a—C6a	1.3420 (19)
N8b—H8b2	0.992 (15)	C6a—H6a	0.93
N8b—C4b	1.3174 (17)	O1—H3	1.223 (14)
H8b1—H8b2	1.64 (2)	O1—C4	1.2793 (16)
C4b—C5b	1.4123 (17)	O2—C4	1.2376 (15)
C5b—H5b	0.93	O3—H3	1.201 (14)
C5b—C6b	1.3373 (18)	O3—C1	1.2789 (15)
C6b—H6b	0.93	O4—C1	1.2354 (15)
O7a—C2a	1.2356 (14)	C1—C2	1.4886 (19)
N1a—H1a	0.952 (14)	C2—H1	0.93
N1a—C2a	1.3591 (15)	C2—C3	1.328 (2)
N1a—C6a	1.3535 (17)	C3—H2	0.93
N3a—C2a	1.3679 (15)	C3—C4	1.4859 (19)
N3a—C4a	1.3493 (15)	H1n3b—H1n3a	1.11 (2)
			
H1b—N1b—C2b	117.2 (8)	H8a2—N8a—C4a	119.4 (10)
H1b—N1b—C6b	120.3 (8)	O7a—C2a—N1a	121.32 (11)
C2b—N1b—C6b	122.48 (11)	O7a—C2a—N3a	121.14 (10)
C2b—N3b—C4b	121.52 (10)	N1a—C2a—N3a	117.53 (10)
C2b—N3b—H1n3b	118.7 (15)	N3a—C4a—N8a	117.68 (11)
C4b—N3b—H1n3b	119.8 (15)	N3a—C4a—C5a	120.20 (11)
H8b1—N8b—H8b2	120.1 (13)	N8a—C4a—C5a	122.12 (12)
H8b1—N8b—C4b	118.2 (9)	C4a—C5a—H5a	121.18
H8b2—N8b—C4b	121.2 (9)	C4a—C5a—C6a	117.64 (12)
O7b—C2b—N1b	121.19 (11)	H5a—C5a—C6a	121.18
O7b—C2b—N3b	121.54 (10)	N1a—C6a—C5a	121.06 (12)
N1b—C2b—N3b	117.28 (10)	N1a—C6a—H6a	119.47
N3b—C4b—N8b	117.51 (11)	C5a—C6a—H6a	119.47
N3b—C4b—C5b	119.83 (11)	H3—O1—C4	114.1 (8)
N8b—C4b—C5b	122.67 (11)	H3—O3—C1	113.3 (8)
C4b—C5b—H5b	121.07	O1—H3—O3	170.6 (15)
C4b—C5b—C6b	117.86 (12)	O3—C1—O4	123.05 (12)
H5b—C5b—C6b	121.07	O3—C1—C2	120.35 (11)
N1b—C6b—C5b	121.00 (12)	O4—C1—C2	116.60 (12)
N1b—C6b—H6b	119.5	C1—C2—H1	114.68
C5b—C6b—H6b	119.5	C1—C2—C3	130.64 (13)
H1a—N1a—C2a	119.2 (8)	H1—C2—C3	114.68
H1a—N1a—C6a	118.4 (8)	C2—C3—H2	114.74
C2a—N1a—C6a	122.28 (11)	C2—C3—C4	130.53 (13)
C2a—N3a—C4a	121.30 (10)	H2—C3—C4	114.74
C2a—N3a—H1n3a	121.9 (18)	O1—C4—O2	122.93 (12)
C4a—N3a—H1n3a	116.8 (18)	O1—C4—C3	119.78 (11)
H8a1—N8a—H8a2	120.2 (14)	O2—C4—C3	117.28 (12)
H8a1—N8a—C4a	120.4 (9)		

### Hydrogen-bond geometry (Å, º)

**Table:**

*D*—H···*A*	*D*—H	H···*A*	*D*···*A*	*D*—H···*A*
N1*b*—H1*b*···O2^i^	0.956 (15)	1.824 (15)	2.7718 (14)	170.8 (13)
N8*b*—H8*b*1···O7*b*^ii^	0.900 (17)	2.030 (18)	2.8517 (14)	151.2 (13)
N8*b*—H8*b*2···O7*a*	0.992 (15)	1.850 (15)	2.8411 (15)	177.4 (12)
C5*b*—H5*b*···O2^iii^	0.93	2.43	3.3347 (16)	164.60
N1*a*—H1*a*···O4	0.952 (14)	1.793 (14)	2.7411 (14)	173.2 (12)
N8*a*—H8*a*1···O7*b*	0.897 (16)	1.959 (16)	2.8555 (15)	179.0 (13)
N8*a*—H8*a*2···O7*a*^iv^	0.885 (17)	2.028 (18)	2.8368 (15)	151.5 (14)
C5*a*—H5*a*···O4^iv^	0.93	2.37	3.2970 (16)	175.15
O1—H3···O3	1.223 (14)	1.201 (14)	2.4155 (12)	170.6 (15)
O1—H3···C1	1.223 (14)	2.071 (15)	3.0775 (15)	136.7 (11)
O3—H3···O1	1.201 (14)	1.223 (14)	2.4155 (12)	170.6 (15)
O3—H3···C4	1.201 (14)	2.100 (15)	3.0927 (15)	137.4 (12)
N3*b*—H1*n*3*b*···N3*a*	0.861 (16)	1.979 (16)	2.8398 (14)	178 (2)
N3*a*—H1*n*3*a*···N3*b*	0.873 (18)	1.970 (18)	2.8398 (14)	174 (3)

Symmetry codes: (i) *x*+1/2, −*y*+3/2, *z*−1/2; (ii) *x*, *y*−1, *z*; (iii) *x*+1/2, −*y*+1/2, *z*−1/2; (iv) *x*, *y*+1, *z*.

## References

[bb1] Becker, P. J. & Coppens, P. (1974). *Acta Cryst.* A**30**, 129–147.

[bb2] Benali-Cherif, N., Falek, W. & Direm, A. (2009). *Acta Cryst.* E**65**, o3058–o3059. 10.1107/S1600536809046571 PMC2972138 21578789

[bb3] Burla, M. C., Caliandro, R., Camalli, M., Carrozzini, B., Cascarano, G. L., De Caro, L., Giacovazzo, C., Polidori, G. & Spagna, R. (2005). *J. Appl. Cryst.* **38**, 381–388.

[bb4] Etter, M. C., MacDonald, J. C. & Bernstein, J. (1990). *Acta Cryst.* B**46**, 256–262. 10.1107/s0108768189012929 2344397

[bb5] Fábry, J., Dušek, M., Vaněk, P., Rafalovskyi, I., Hlinka, J. & Urban, J. (2014). *Acta Cryst.* C**70**, 1153–1160. 10.1107/S2053229614024632 25471417

[bb6] Groom, C. R. & Allen, F. H. (2014). *Angew. Chem. Int. Ed.* **53**, 662–671. 10.1002/anie.201306438 24382699

[bb10] Nonius (1998). *KappaCCD Server Software*. Nonius BV, Delft, The Netherlands.

[bb7] Otwinowski, Z. & Minor, W. (1997). *Methods in Enzymology*, Vol. 276, *Macromolecular Crystallography*, Part A, edited by C. W. Carter Jr & R. M. Sweet, pp. 307–326. New York: Academic Press.

[bb8] Petříček, V., Dušek, M. & Palatinus, L. (2014). *Z. Kristallogr.* **229**, 345–352.

[bb9] Spek, A. L. (2009). *Acta Cryst.* D**65**, 148–155. 10.1107/S090744490804362X PMC2631630 19171970

